# Tracking health sector priority setting processes and outcomes for human resources for health, five-years after political devolution: a county-level case study in Kenya

**DOI:** 10.1186/s12939-020-01284-3

**Published:** 2020-09-21

**Authors:** Joshua Munywoki, Nancy Kagwanja, Jane Chuma, Jacinta Nzinga, Edwine Barasa, Benjamin Tsofa

**Affiliations:** 1 KEMRI Wellcome Trust Research Programme, KEMRI Centre for Geographic Medicine Research Coast, Kilifi, Kenya; 2 Department of Public Health, School of Human and Health Sciences, Pwani University, Kilifi, Kenya; 3 The World Bank Group, Kenya Country Office, Nairobi, Kenya

**Keywords:** Priority setting, Health system decentralisation, Human resources for health, Decision space

## Abstract

**Background:**

Health sector priority setting in Low and Middle-Income Countries (LMICs) entails balancing between a high demand and low supply of scarce resources. Human Resources for Health (HRH) consume the largest allocation of health sector resources in LMICs. Health sector decentralization continues to be promoted for its perceived ability to improve efficiency, relevance and participation in health sector priority setting. Following the 2013 devolution in Kenya, both health service delivery and human resource management were decentralized to county level. Little is known about priority setting practices and outcomes of HRH within decentralized health systems in LMICs. Our study sought to examine if and how the Kenyan devolution has improved health sector priority setting practices and outcomes for HRH.

**Methods:**

We used a mixed methods case study design to examine health sector priority setting practices and outcomes at county level in Kenya. We used three sources of data. First, we reviewed all relevant national and county level policy and guidelines documents relating to HRH management. We then accessed and reviewed county records of HRH recruitment and distribution between 2013 and 2018. We finally conducted eight key informant interviews with various stakeholder involved in HRH priority setting within our study county.

**Results:**

We found that HRH numbers in the county increased by almost two-fold since devolution. The county had two forms of HRH recruitment: one led by the County Public Services Board as outlined by policy and guidelines and a parallel, politically-driven recruitment done directly by the County Department of Health. Though there were clear guidelines on HRH recruitment, there were no similar guidelines on allocation and distribution of HRH. Since devolution, the county has preferentially staffed higher level hospitals over primary care facilities. Additionally, there has been local county level innovations to address some HRH management challenges, including recruiting doctors and other highly specialized staff on fixed term contract as opposed to permanent basis; and implementation of local incentives to attract and retain HRH to remote areas within the county.

**Conclusion:**

Devolution has significantly increased county level decision-space for HRH priority setting in Kenya. However, HRH management and accountability challenges still exist at the county level. There is need for interventions to strengthen county level HRH management capacity and accountability mechanisms beyond additional resources allocation. This will boost the realization of the country’s efforts for promoting service delivery equity as a key goal – both for the devolution and the country’s quest towards Universal Health Coverage (UHC).

## Introduction

Priority setting is a key health sector management function that entails balancing between a high demand for scarce resources and their efficient allocation [1]. Human Resources for Health (HRH) are argued to be a critical component of a health system that is also a large consumer of health sector resources [2–4]. Even with the highest allocation from their respective health sector budgets, most countries still face a chronic HRH shortage in addition to spatial and skillset maldistribution [3, 5]. This scarcity is more prominent in low and middle-income countries (LMICs), with most of the countries having a less-than-critical workforce density being in sub-Saharan Africa, a region with only 3% of the global health workforce (less than 22.8 health workers per 10,000 population) and yet bears 25% of the global burden of disease [4, 5]. Kenya is among the countries identified as HRH crisis countries since it does not have sufficient numbers of health workers to meet the threshold density ratio (number of health workers needed to adequately cover the population with essential health services) [4].

Decentralization is a health sector governance reform that has been adopted in many LMICs due to its perceived utility for increasing public participation and accountability in the management of public resources as well as potential to increase management efficiency over public resources [6–9]. In 2013, Kenya adopted a devolved government system in order to address historical equity concerns in regional resource allocation and increase efficiency and accountability in the management of public resources [8]. This devolution led to increased county-level decision space and control over the management of health sector resources, including HRH [2, 10]. One study outlining the institutionalization of formal and informal accountability in decentralized health systems found these lines of accountability to be influencing decision making over health sector resources at the county level in Kenya [11]. It is thus of great importance to critically understand and maximise HRH priority setting in decentralised health settings for the various reasons outlined above.

Under Kenya’s devolved governance, county governments are responsible for health service delivery, including human resource management, while the national government undertakes pre-service training and policy formulation [10]. The county governments are made of two main arms. First is the executive arm comprising of an elected Governor, Deputy Governor and ten members of the County Executive Committee (CEC) that represent each of the ten county government departments, including the County Department of Health (CDoH). The CEC members are appointed by the Governor [12]. The second arm is the County Assembly which is the legislative arm made of Members of County Assembly (MCAs), who are elected to represent electoral wards, and some reserved seats of nominated members to represent special interest groups. The nominated members are nominated by political parties based on their respective party numerical strengths from the elected members. In addition, there is a semi-autonomous County Public Services Board (CPSB) that has the overall management and oversight role for the management of all county government employees. The CPSB members are appointed by the Governor with approval from the County Assembly. However, once constituted, it is legally mandated to operate independent of both the County executive and County Assembly.

The Kenyan health system is organized into six levels of service delivery with Levels 1–5 being managed by county governments. Level 1 facilities (community health units) are responsible for community level services. Level 2 facilities (dispensaries) and Level 3 facilities (health centers) are responsible for primary healthcare (PHC) services, particularly basic outpatient services and referrals. Level 4 (sub-county and county referral hospitals) and Level 5 (regional referral hospitals) offer specialized outpatient and comprehensive inpatient services. Level 6 (national referral hospitals and other national referral services) offer highly specialized healthcare and are managed by the national government [13]. In 2017, after the general election held late that year, the country embarked on an ambitious political journey for attaining Universal Health Coverage (UHC) for all its citizens by the year 2020 as part of the government of Kenya’s big-four agenda [14].

Most meso-level priority setting studies in LMICs focus on priority setting practices, with little focus on priority setting outcomes [1, 6]. In addition, in spite of many LMICs adopting decentralisation reforms, very little is known about health sector priority setting outcomes within decentralised settings in LMICs, and no study had been done to examine county level HRH priority setting processes and outcomes in Kenya since the 2013 devolution [1]. This paper seeks to contribute to filling these two gaps in literature.

## Study methods

### Study design

We undertook a mixed methods case study in one of the 47 counties in Kenya. We purposefully selected one county to allow for more detailed and in-depth exploration of health sector priority setting and devolution, both of which are complex phenomena for health system organisation and functioning [15, 16]. For this study, we employed multiple data collection methods including documents review, records review and Key Informant Interviews (KIIs) with an aim to triangulate findings and increase rigour [17]. We used HRH as a tracer element of tracking health sector priority setting for the 2013–2018 devolution period [2].

### Study setting

We conducted the study in one of the six counties found in the Kenyan Coast. We purposefully selected this county partly because of its proximity to our research institution and partly because of the long-term and close working relationships we have had with various county-level health system managers there, which allowed us ease of access to data and information that wouldn’t be necessarily made available without these long-term relationships founded on trust building. This is a common methodological consideration for health policy and systems research projects, especially those seeking to examine health system governance issues that are often considered “politically” sensitive [18, 19].

### Study conceptual framework

**Fig. 1:**
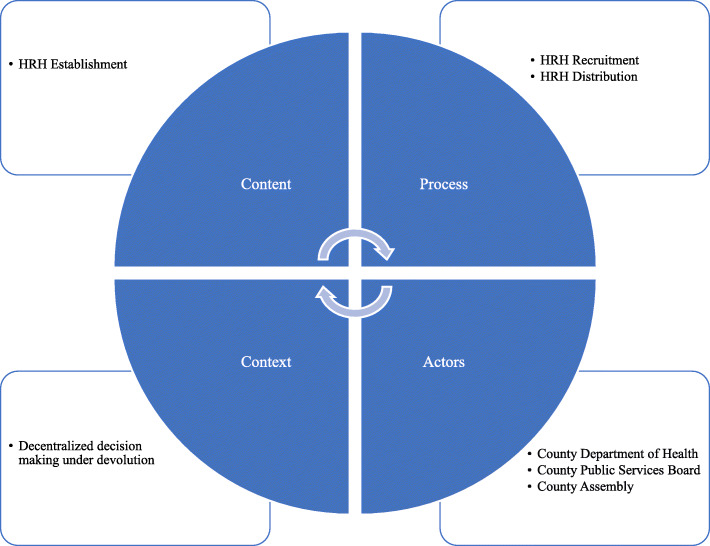
Study Conceptual Framework

For this study, we applied the policy analysis triangle proposed by Walt and Gilson [20]. In this framework the authors argue that (health) policy is an “an outcome of complex social, political and technical interactions.” Therefore, analysis of a policy should not only focus on the content but also look in to the context, process and actors involved in the process of its development and implementation [20]. In applying this framework on our study, *content* refers to the HRH establishment, which is the HRH cadres recruited within the county after devolution and the level of care they have been deployed to. *Context* refers to Kenya’s devolved health system in which priority setting for HRH is happening at the county level and the study county political context. *Process* refers to how recruitment and distribution of HRH has been happening by cadre and by level of care. Finally, *actors* are decision-makers at management level for the different cadres and levels of care involved in the recruitment and distribution of HRH. Figure 1 is a summary of the adopted conceptual framework.

### Data collection procedures

We collected and/or assembled data from three sources namely: review of official national and county government documents; official county government HRH records; and interviews with key informants. For official government documents we reviewed all legal, policy and guideline documents that touched on the management of HRH at county level (Table 1). We summarized data from these documents using a content extraction tool. We developed this content extraction tool guided by our study objectives and study conceptual framework.

**Table 1:** Legal, policy and guideline documents on HRH management reviewed

No.	Document Reviewed
1	County Government Act 2012
2	Public Services Commission Human Resource Manual
3	Kenya Health Policy 2014–2030
4	Human Resources for Health Norms and Standards Guidelines for the Health Sector
5	Devolved Human Resource Management Policy Guidelines

For the records review, although we initially intended to use records from the CDoH, on accessing these, we realised that the HRH recruitment and deployment records held by the CDoH were incomplete and had inconsistencies – especially for the early years of devolution. We, therefore, accessed and utilized the data from the overall county government master pay-roll held at the County Department of Public Services to extract the HRH data. We used the payroll data of December each year between 2013 and 2018 to estimate the total number of health workers and new recruitments that the county had annually since 2013. We used a table extraction form to extract data from the county master pay-roll database and interpreted the annual increments in payroll HRH numbers to mean new HRH recruitments each year (though continuous of natural attrition). We validated the payroll numbers by checking for concurrence with the managers we interviewed.

Finally, JM and NK conducted eight interviews with Key Informants involved in the recruitment, distribution and general HRH management in the county government. These were drawn from the CDoH at county, sub-county and facility levels; from the County Department of Public Services, and from the CPSB. We were unable to get an interview appointment with a sitting Member of County Assembly (MCA) even after several attempts of trying to schedule this. For all the interviews, we used an interview guide that we had developed with questions guided by our study objectives, our conceptual framework, themes from literature review, documents review findings and records review findings. We obtained informed consent for all interviews, audio recorded them and later transcribed verbatim.

### Data management and analysis

We kept audio records and HRH payroll data under lock and key at all times and after transcription of the audio records, the transcripts were saved in a password-protected computer. The digital formats of the HRH audio records and recruitment/distribution data were stored in an H-Drive provided by the research institution, which only we the researchers could access.

We used a framework analysis approach for our qualitative data analysis [21]. To do this, we first developed key themes and sub-themes using our study objectives and conceptual framework. After transcribing KIIs, we read through all the transcripts to look for additional emerging themes and used these to refine our initial thematic frame. We then imported the interview transcripts and document review content extraction summaries into N-Vivo 9 software for coding and charting.

For quantitative data, we downloaded all the data from source databases into excel spreadsheets. We then used STATA 12 software to do a descriptive analysis of the HRH recruitment and deployment data.

#### Ethical considerations

The study was reviewed and approved by the Kenya Medical Research Institute (KEMRI) Scientific and Ethics Review Unit (SERU) – Ethical approval reference number KEMRI/RES/7/3/1.

#### Study results

In this section, we provide a brief overview of how HRH priority setting used to happen prior to devolution, followed by findings on how HRH priority setting process now happens after devolution, both in theory and practice within our study county. Finally, we present the outcomes of devolved HRH priority setting in our study county.

**Table 2:** Summary of Devolved HRH Priority Setting Practices in Theory and Practice at the Study County

HRH Priority Setting Function	Theory	Practice
**HRH Recruitment**	The CPSB was responsible for HRH recruitment. Both the CPSB and the CDoH were supposed to work jointly in the recruitment process – from identification of HRH needs to confirmation of new staff.	The county had two parallel recruitments: a formal HRH recruitment done jointly by the CPSB and CDoH, and a parallel, politically driven recruitment done and managed by the CDoH without involvement of the CPSB as required by the law.
**HRH Distribution**	There were no guidelines on which institution was responsible for HRH distribution.	The CDoH was responsible for HRH distribution and used a concept of ‘bare minimum’ to determine how many HRH to deploy to a given facility. The CDoH had also started implementing the incentives guidelines to attract more HRH to the less attractive and rural primary healthcare facilities

## HRH priority setting in Kenya prior to devolution

Prior to devolution, the national Public Service Commission was responsible for recruitment and deployment of all public servants including HRH. The commission delegated HRH management to the national Ministry of Health (MoH). HRH recruitment and deployment were done centrally by the national MoH, and decisions over the distribution of health workers across the country were also determined from the national MoH. Districts (and later counties) had very minimal role in determining the number and type/cadre of health workers they would receive from the national deployment [10].

## HRH priority setting at the county level under devolution

### HRH priority setting in theory

From the review of policy and legal documents accessed, devolved HRH recruitment should be a joint responsibility of the County Public Services Board (CPSB) and the County Department of Health (CDoH). The recruitment process should begin with identification of staffing gaps by respective heads of divisions within the CDoH and respective health facility managers [22]. These gaps are to be drawn based on the organizational structure of the CDoH, health facility staffing norms, the schemes of service of various HRH cadres, and health worker career progression guidelines [23]. The Chief Officer of Health and the Human Resource Manager at the CDoH (who is seconded from the County Department of Public Service but based in the CDoH) then consult the CPSB for approval of the HRH vacancies identified to be filled [12, 22]. To approve the declared vacancies, the CPSB would seek to verify the number of vacancies identified, when they occurred and whether the vacancies are within the authorized establishment for the CDoH. The CPSB further consults with County Treasury to ascertain that the CDoH has the necessary required budgetary allocation to fill up the identified vacancies [22].

The CPSB then advertises the declared and approved vacancies for a period of at least 3 weeks via various media outlets and in the communities through administrative channels so that the marginalized communities are reached as well. All interested applicants have to fill a prescribed application forms and submit to the CPSB [22].

The CPSB in liaison with the Chief Officer of Health develop a short-listing criterion as guided by relevant legal and policy requirements for the positions to be filled. The CPSB is then required to counter check with relevant professional bodies to ensure that the shortlisted HRH candidates are all duly and appropriately registered. Short-listed candidates are then invited for interviews through the media outlets [22].

Candidates for the different positions should be selected based on merit, fair competition and representativeness of the diversity of the county [12]. The board coordinates and monitors the recruitment process to ensure equity and transparency [22].

Final candidates for the respective positions are rationalized and approved by the CPSB. The CDoH’s Human Resource Manager then prepares appointment letters with terms of service, which are then signed by an authorized officer, who can be from the CPSB or to whom the CPSB has delegated its authority. The CDoH’s Human Resource Manager should then communicate with the appointed candidates to pick their appointment letters [22].

The recruited staff can accept or reject the offer in fourteen (14) days. After 14 days, the Human Resource Manager should advise on how to fill the resulting vacancies in case any of the new recruits rejects the job offer. Officers that have accepted their appointment should be put on probation for 6 months, after which if their performance be satisfactory, they should be confirmed and admitted in to the permanent and pensionable establishment by the public service board [22] unless they are employed on contract terms.

Fixed-term contract employments are either medium-term or short-term. Medium term contracts run for a maximum of 5 years and are subject to one renewal whereas short-term contracts cannot be engaged for more than 3 months. Casuals workers can be engaged on urgent, short-term contracts by the CDoH, with approval of the CPSB [22].

HRH recruited by the donor contractors should also be informed by the CDoH HRH needs and the workers paid as per government guidelines. If there is an agreement between the donor and government, the donor workers get absorbed at the end of the contract as per the agreement [22].

From the review of policy and legal documents, it is not clear which institution of office within the county government has the ultimate responsibility for distribution and deployment of health workers.

### Devolved HRH recruitment in practice and its influences

Since its establishment in 2013, our study county had been recruiting health workers through two parallel mechanisms. One of them is led by the CPSB as per the existing policy and legal requirements. However, there has existed another process where health workers dubbed ‘casual workers’ are recruited directly by the CDoH on short-term contract without the involvement or participation of the CPSB.

At the end of each financial year, sub-county health management teams and hospital management teams do submit their HRH requirements to the County Health Management Team (CHMT), which is the senior management organ of the CDoH. At the same time, the CDoH human resource unit establishes transitions that have occurred in that particular year i.e., deaths, transfers, resignations and retirements.

CM002: *“In anything, you must start from the user. So the user can be in most cases be it the hospital or be it us a sub-county. So we make these requests through the {CDoH} as a team or as respective {cadre or sub-county or hospital}. … so the different needs from different hospitals and sub-counties are submitted to the county.”*

At the CDoH, a human resource advisory council was established consisting of the County Director of health, CEC Member for Health, Chief Officer of Health, representatives of core cadres such as the doctors, nurses and clinical officers; and the Human Resource Manager. This council does sit to look at human resource issues raised more holistically; it looks at the raised requests against available HRH finances and deliberates whether the submitted requests could be fulfilled. The advisory council then advises the CHMT based on their findings, after which the CDoH submits their HRH request to the CPSB.

Upon receiving the request, the CPSB also looks into the laws that guide the recruitment process and engages Chief Officer of Finance to ascertain the budgetary allocation of the CDoH and affordability of the requested new recruits by the department.

After approving the recruitment request from the CDoH, the CPSB undertakes the hiring process on behalf of the CDoH. The CDoH Human Resource Manager undertakes a technical role in the recruitment process - including taking part in the shortlisting and interviewing activities led by the CPSB. Once the new staff are hired, have received appointment letters and reported to the Human Resource Manager, the posting and deployment of these new staff is undertaken by the Chief Officer of Health.

From the interviews, the key influencing factors for health worker recruitment at the county are largely (i) push and demands from local politicians to create jobs for “their people,” (ii) service need owing to opening of new health facilities, and (iii) budgetary limits set to the CDoH over HRH expenditure.

Interviewees reported that local politicians have over the time used their influence to have “their people” employed by the CDoH, including those without necessary qualification. It was however reported that the CDoH human resource unit and the CPSB had been resisting to recruit workers that do not meet minimum qualification as per the scheme of service. The politicians then began circumventing the process of recruitment through the CPSB and compelled the CDoH to create a parallel recruitment for HRH as short-term casual employees. These “casual workers” (largely proposed by local politicians) also included health professionals who would be hired on short-term contract and managed by the CDoH without involvement of the CPSB as required by legal and policy provisions. Unlike the CPSB formal employment, no advertisements were made for these casual workers.

CM004: *“Like now, here {*one of the local dispensaries*} … , when they wanted staff, the MCA {*local Member of County Assembly*} brought 7 casuals to go there … and in a dispensary, we are not supposed to have more than 3 casuals, i.e. a gardener, a cleaner and a watchman”*

Due to the political and emotive nature of the casual workers, their recruitment and deployment was thus handled directly by the senior managers of the CDoH. At some point, the CDoH made a request to have the contracted ‘casuals’ absorbed in to permanent employment by the CPSB. The CPSB declined to employ them as they could not obtain a justification for their employment. However, politicians continued to pile pressure on the CPSB, pushing it to absorb these ‘casuals’ that had been recruited without their involvement. The CPSB eventually absorbed the ‘casuals’ who had the requisite qualifications in to the permanent and pensionable scheme. However, most of the casual workers who had been informally recruited did not meet minimum qualifications and thus could not be absorbed.

CM004: *“we wrote a memo, we have to go through their papers. So, we went and applied and we verified their things. We took 24, and the rest … they were told in advance that after 3 months, you’re no longer going to, you have to reapply.”*

In the early days of devolution, there was a political push to open new health facilities. The CDoH would then use these new facilities as a basis for obtaining political goodwill from the MCAs to hire more health workers. The corresponding increase in HRH and facility numbers, however, did not help address existing chronic health worker shortage in the county.

CM002: *“We’ll tell the MCA, okay, we’ve opened {the facilities}. I know you want services for your people, but look at this. We now have one person seeing this population. (S) he has no replacement/substitute. If the person falls sick today, who will come?”*

The CDoH had a budgetary ceiling of 30% of its budgetary allocation to salaries and other remunerations and some managers acknowledged that the county was currently at the ceiling of its HRH recruitment budget. It thus had capacity to replace HRH but not to employ more, unless the ceiling was lifted, or more funds were allocated for HRH salaries and remuneration.

### County level HRH deployment in practice, and its influences

From the interviews, it was reported that the county does not have set guidelines on distribution of HRH. Given the scarcity of HRH in the county, the distribution of employed staff has been guided by the concept of bare minimum in distributing health workers in the county i.e., the minimum number that each facility is supposed to have. The staff postings are usually done by the Chief Officer at the CDoH after consultation with key managers.

CM001: *“Right now we are one thousand, five hundred and fifty-one {1551} health workers and that number is still very low. In fact, it is the bare minimum number in every place. And it’s like half of the population of the county.”*

Whenever the CDoH got new staff, top managers would sit down and deliberate on what they had. The managers consider factors such as HRH requests made and workloads of health facilities. Heads of respective HRH cadres had a big influence over the distribution of the respective cadres given that they were responsible for the services provided by those particular cadres. Deployment from the county level would be done to the county hospitals and sub-county health management units. The sub-county health management units would then determine factors such as current staff numbers and workloads in the respective PHC facilities, then subsequently distribute the HRH they receive to their PHC facilities.

It was reported that occasionally, some staff would be deployed/re-deployed for disciplinary reasons i.e., staff considered to be undisciplined would be transferred from rural facilities and closer to where managers are based for easier monitoring of their conduct.

Table 2 summarizes the county-level health sector recruitment and deployment roles both in theory and in practice.

## HRH priority setting outcomes at the Study County under devolution

### HRH recruitment outcomes

**Fig. 2:**
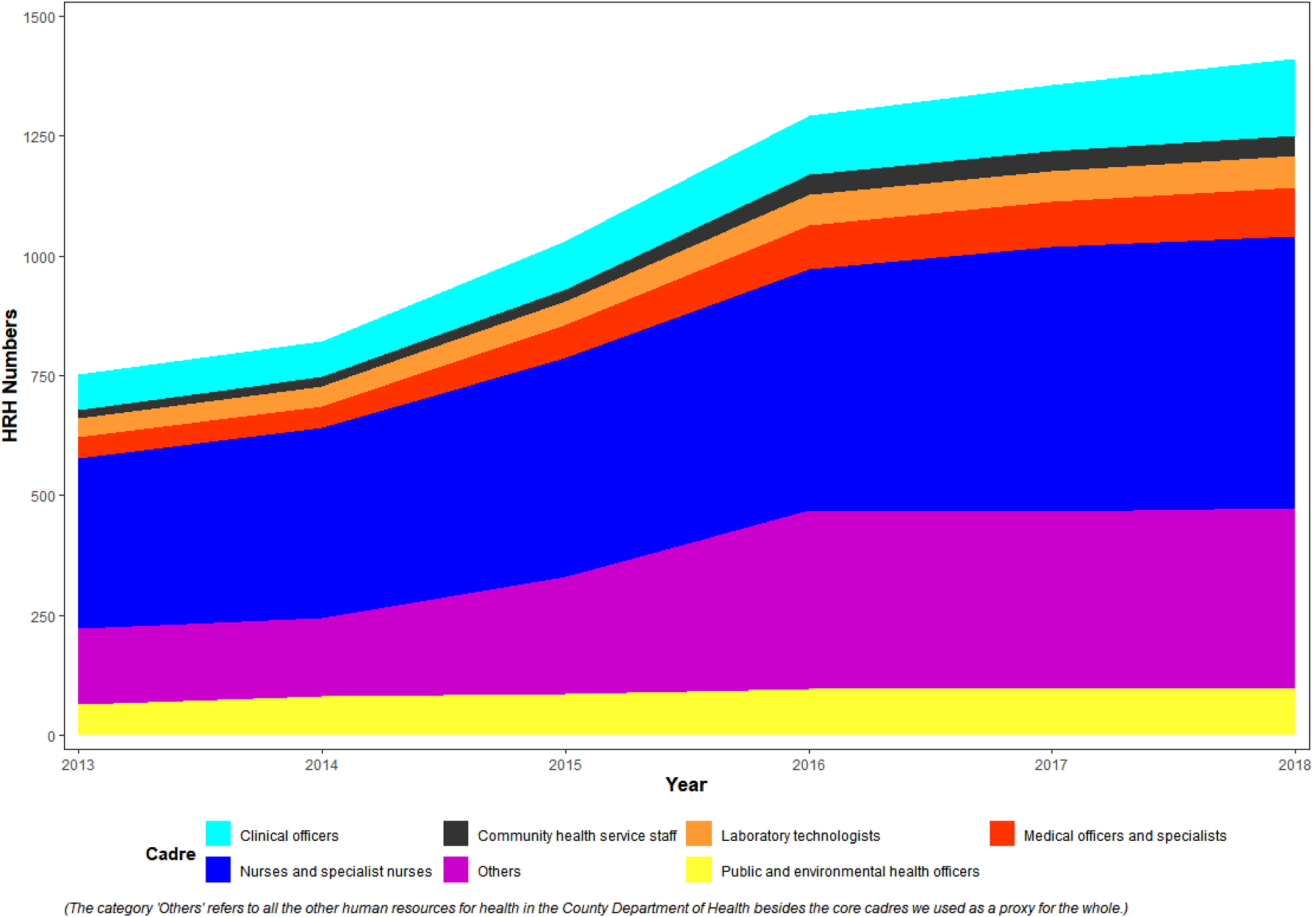
Devolved HRH Recruitment by Cadre in the Study County, 2013–2018

Figure 2 shows how HRH numbers at the study county have changed between 2013 at devolution and 2018 based on payroll data. The total HRH numbers almost doubled (increased from 752 to 1412). However, even with the increase in numbers of health workers recruited, from the interviews, it was reported that the county still did not have adequate numbers of HRH it required to provide services in the health facilities within the county.

CM006: *“ … like for example, a dispensary is supposed to have 4-6 nurses – that is the norm … but to my subcounty that one has never happened. Because the nurses are few … I have one dispensary which is currently being run by one nurse and the rest of the dispensaries have two nurses each … ”*

CM007: *“ … you find one nurse at the same time having three deliveries. And she’s all alone. So they suffer burnout … ”*

From the records, nurses were consistently the highest recruited cadre of health workers whereas community health services staff were the least recruited. Based on the KIIs, the county has been prioritizing the recruitment of nurses and clinical officers as they are the main cadres required, both in PHC facilities and in the referral facilities.

### HRH distribution outcomes

**Fig. 3:**
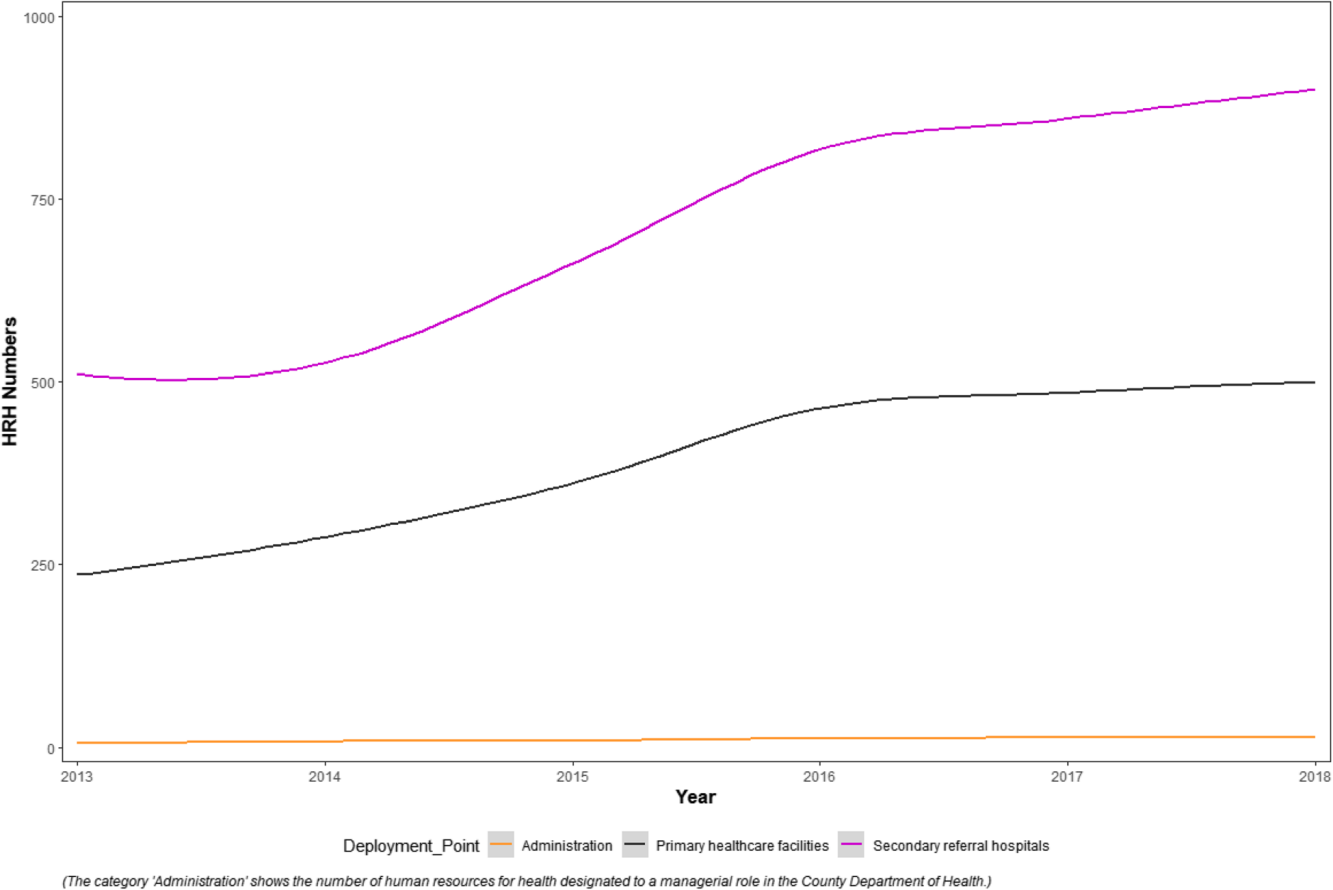
Devolved HRH Deployment by Level of Care in the Study County, 2013–2018

Figure 3 shows the distribution of CDoH HRH to either hospitals (Level 4 facilities), primary healthcare (Level 2 and 3 facilities) or administration. No data is presented on community health units because we learned from the interviews that though community health assistants were based in the communities, they were counted and managed under respective facilities in the county. The category “administration” shows the number of CDoH staff serving in the administrative capacity at the different management levels.

From the records, and as illustrated in Fig. 3, the county has been prioritizing deployment of health workers to hospitals (secondary care) levels as opposed to PHC facilities since devolution. PHC facilities, which are also located in rural setups, also reported that they would occasionally lose some of their staff to referral hospitals; when PHC staff went to study and came back with specialized training, they were considered to be better placed in referral facilities and thus posted there.

From the interviews, the decision to prioritize the deployment of newly employed staff to hospitals by the CDoH leadership was because Level 4 facilities are the referral facilities for PHC facilities; The interviewees argued that the CDoH intended to first improve service delivery in the level 4 facilities, before focusing on primary health facilities.

CM002: *“ … so the main idea has been to improve services here {county referral facility}. Basically, it’s a whole approach but it’s trying to improve the other much better or faster because we’ll be failing if someone has gone to primary healthcare and missed the specialized care and they come here {county referral facility} and they miss it. Then what are we doing?”*

### Other outcomes of devolved HRH priority setting

Interviewees reported that in the early days of devolution, the county government would employ doctors to address a staffing shortage and 2 years down the line, the doctors would take a long study leave leading - to a pseudo-shortage. Given that the medical doctors were scarce at the county level and their salaries quite high compared to the rest of the cadres, it was expensive for the county to keep paying the doctors on study leave while also paying new doctors recruited to fill the re-introduced staffing gap. The county government thus resolved to employ medical doctors and specialists on fixed-term contract basis as opposed to the permanent and pensionable basis used for the rest of the (lower cadre) staff.

CM002: *“ … the county used to absorb doctors and after every two years, people used to go and study. So we (*had*) said (*that*) we had a gap, we get {*doctors} *then a year or two you go (*then*) we’re back there (*to the staffing gap*) … so then it’s like a wheel. You get people, you say you are fine but the next day they are gone (*and now*) you are not fine … ”*

To better attract and retain health workers, the county developed and began implementing a new health worker incentive guideline in 2018. The CDoH now includes health worker awards and recognitions costs in its budget. This was particularly necessary for PHC facilities that were in rural settings and did not have attractive working environments - rural setups are underdeveloped and marginalized and the interviewed managers reported that health workers did not find working in PHC facilities to be attractive.

## Discussion

In this section, we begin by presenting a summary of our findings. We then proceed to interrogate and discuss our findings while applying the decision space framework as originally developed by Bossert (1998), and applied by Bossert and Mitchell (2011), and by Tsofa el al (2017) [10, 24, 25].

In summary, our study found that, since devolution, HRH numbers have increased almost two-fold at the county, though these numbers were still reported to be inadequate due a corresponding increase in numbers of health facilities during the same period. The county had two forms of HRH recruitment, one led by the CPSB as required by policy and guidelines, and another parallel, politically-driven one done directly by the CDoH. HRH allocation and distribution were mainly guided by the ‘bare minimum requirements.’ And though there were clear guidelines on HRH recruitment, there were no policy guidelines on HRH allocation and distribution. As a result, the county preferentially staffed higher level hospitals over primary care facilities. Additionally, the county initiated local interventions - including recruiting doctors and highly specialized staff on fixed term contract basis - and implemented local incentives for attracting and retaining HRH to remote areas.

In one of the early studies analysing health system decentralisation, Thomas Bossert, drawing from the *principal-agent-theory*, developed the decision space framework [24]. This framework and its subsequent improvements have continued to find wide application in many studies on health system decentralisation. In a 2011 publication, Bossert and Mitchell suggested further improvements of the decision space frame-work by arguing that the outcomes of health system decentralisation is not only affected by what decisions have been decentralised (decision space) but also by the organisation structure and capacity of the decentralised units; and the accountability arrangements [25]. This improved framework has more recently been applied by members of our group in analysing the health sector effects of the early days of implementation of devolution in Kenya [10].

In applying the Bossert and Mitchell framework on our findings; we see an increase in decision space at the county level when the devolved government system decentralised a certain number of HRH management functions to the county governments. The devolved functions include HRH recruitment and distribution, promotions, disciplinary actions, trainings and management of HRH payroll. Decentralization brought decision-making over HRH priorities closer to the people and with the increased decision space, the county has used the increased autonomy to recruit more HRH – mostly nurses and clinical officers, who are the main cadres in both secondary and primary healthcare facilities. The CDoH is accountable locally to the county executive, with senior CDoH managers being appointed by the Governor and approved by the County Assembly [2, 12].

Within the devolved structure, the national government has maintained oversight, policy formulation and capacity building roles. These include development of overall HRH management policies and guidelines. Decentralised health system structures and how they make decisions over HRH investments and management has been shown to have various similarities and differences across different countries [26]. For instance, Tanzania has been reported to have district boards that are charged with local HRH recruitment, similar to the CPSB in Kenya [27]. A study in India on the other hand reported presence of a different decentralized structure that consists of states as decentralized units and districts as sub-units within the states, with recruitments happening through the District Health Societies [28]. In another study from Mozambique, it was reported that at the provincial level, which was the decentralized unit, the provincial Governor was responsible for HRH management but could delegate responsibility to provincial directors [9]. A common thread across these studies is that despite the different decision-making processes, health system decentralization brought HRH management decision-making to a decentralized unit of governance that is closer to the people.

Our study shows that the county has been learning from its management challenges and progressively improving its management capacity since devolution. Challenges reported in earlier studies like the lack of clarity over HRH responsibilities of the CDoH and CPSB were addressed through the creation of the office of Human Resource Manager seconded from the CPSB to the CDoH [10]. The office serves as a bridge between the CDoH and CPSB, helping address the earlier reported ambiguity. The CDoH also currently has a HR Advisory Council that holistically looks at HRH issues and then advises the CHMT, arguably leading to better HRH priority setting. In 2018, the county began implementing the new locally developed Human Resource Incentive Guideline, which is expected to improve the earlier reported challenge of staffing rural PHC facilities as they are less attractive to HRH. This adds to the range of locally generated solutions to local problems that can only be made possible within decentralised decision making and resource management.

Several key informants reported existing HRH challenges such as inadequate HRH numbers despite doubling of HRH numbers since devolution, which could partly be attributed to a corresponding increase in the number of health facilities opened within the county since devolution. This HRH shortage is however not unique to our study county but affects Kenya in general as indicated in a recent study that estimated HRH numbers in all Kenyan facilities (public, private and faith-based facilities) to be 22.7% of the required HRH numbers for effective health service delivery [29]. Additionally, Kenya and other sub-Saharan African countries have constantly reported severe shortages of the required HRH [30].

Other system capacity challenges faced by our study county included: the lack of clear HRH deployment and distribution guidelines that would guide equitable distribution of available HRH resource, and challenged quality of data in the CDoH that potentially affects the use of information to inform devolved HRH priority setting. The issue of poor HRH data quality has also been reported in other countries, and there is need for LMICs to not only improve the quality of records but also strengthen analytical and quantitative skills that would enable better use of evidence to inform decisions [9, 26].

One of the overall devolution goals in Kenya was to improve local accountability over the management of public resources [7, 8]. Early post-devolution studies however reported that the structures established following devolution had limited avenues for public participation, with compromised community participation & accountability as well as the public losing local accountability to the county executives [7, 16, 31, 32]. For instance, an earlier study reported that the vetting and public participation in the recruitment of senior government officials as required by law was conducted merely as a public relations exercise as there was limited capacity for the relevant structures to undertake this excercise [10]. Our study findings also show that the stringent accountability capacity of the CPSB led to its ability to resist political interferences, hence leading to minimal political interference over the formal HRH recruitment process. This, however, was watered down when the local politicians and executive, exerting their political influence, decided to pressurise the CDoH to set up a parallel HRH recruitment process so as to by-pass the accountability to the CPSB. This happened because within the devolved government, local health managers are accountable to local political leaders and the overall politically constituted county excetutive [33]. The politicians themselves feel the pressure and obligation to meet demands from local voters while also having a governing authority over the health sector [34]. This could partly explain why the politicians in our study county influenced the CDoH to manage a parallel recruitment of HRH contrary to the law. Political interference over HRH management has also been observed in Tanzania where local politicians often pushed for their interests in the recruitment and management of HRH [27]. A review also reported that Uganda and Papa New Guinea faced an issue of poor quality staff owing to tribalism and nepotism as well [30].

Within the broader devolution context, our study established that the existing budgetary limits for HRH, lack of HRH distribution guidelines, infrastructural challenges of rural facilities and political interests all influenced the interactions of the devolved decision space, existing accountability mechanisms and organizational capacity hence affecting the overall HRH priority setting outcomes. Some of these contextual factors are not entirely due to devolution but rather unresolved issues from pre-devolution era. A good example is how our study county had already exceeded its staff salaries budgetary ceiling not just because of increased HRH recruitment but also out of obligation to honor delayed promotions and collective bargaining agreements signed pre-devolution [10, 35]. These inherited problems from the pre-devolution era have utilized more of the CDoH budget that would have helped address HRH scarcity, consequently creating a limitation of how many HRH they can employ [33]. The county adapted to this limitation by changing the terms of employment for specialized workers so that they were employed on contract terms, making it easier to replace them when they opt to go for further studies without incurring additional costs of paying an absentee specialist. This local intervention has been seen in other countries where countries use fixed term contract employment as a cost containment measure [30].

Though our study could not ascertain the exact rationale and value for a reported corresponding increase in health facilities even as the county recruited more health workers after devolution, political interests and considerations cannot be fully ruled out considering the broader political context in Kenya. Earlier studies have reported incidents of devolved county governments having high appetite in health sector capital investment for political expediency [8, 10]. Our study findings also concur with these earlier studies as the opening of new PHC facilities was not matched with prioritized deployment of health workers to PHC facilities as shown in the HRH distribution data.. Additionally, HRH recruitment data reveals that under devolution, health workers offering services at the community level were among the least recruited cadres despite their necessity for strengthening health service delivery at community health level. This way, the essence of bringing primary healthcare closer to the people was being overlooked. Investing in primary healthcare would be advantageous as these facilities can handle conditions requiring less attention and care, leaving more complicated conditions to hospitals [26]. Most PHC facilities are however found in rural setups that have geographical and infrastructural challenges that make them less attractive to HRH [9, 27, 36]. Therefore, as decentralized units seek to strengthen PHC staffing, they should also address factors that make rural areas unattractive to staff.

Our study has one major limitation. The decision to purposively focus on one case study county limits certain generalizability aspects of our findings [17]. However, the single county focus provides better opportunity for more depth in bringing out the contextual issues that are key in influencing how the complex phenomena of health sector decentralization plays out.

## Conclusion

Human resources for health constitute one of the largest expenditure items for health sector budgets in many countries. For this reason, a prudent priority setting practice for HRH in any health system is not only a good governance practice, but could also assist health systems to maximise the utilisation of the ever-scarce resources. To achieve this, Kenya needs to continue improving capacity through measures such as developing HRH distribution guidelines that promote staffing of PHC facilities and enhancement of accountability mechanisms so as to reduce political interference over HRH priority setting. Given the close relationship between HRH and health service delivery, more studies should be done on how the two align to further inform HRH priority setting for improved health service delivery at the decentralized level. Beyond health, the working and living environments of rural setups need to be improved so as to make them attractive for HRH working in rural facilities.

From our study findings we see that the Kenyan devolution has significantly increased county level decision-space for HRH priority setting. This has resulted in county-level HRH management decisions matching local needs, innovations such as creation of a Human Resource Advisory Council, and a dedicated human resource management office to address HRH challenges. However, county level accountability and HRH management capacities are still sub-optimal, thus affecting the outcomes of HRH priority setting processes. For policy and practice, we do recommend that beyond additional resource allocation; there is need to strengthen county-level accountability mechanisms and HRH management capacities if the country’s dream for attainment of Universal Health Coverage (UHC) by the year 2020 is to be realized. In addition, we believe that though our study focused on one county, our study findings provide critical insights in the understanding of the complex nature of decentralized health sector priority setting which has an overall implication on availability and equitable distribution of health services; both of which have a bearing on the country’s’ efforts and progress toward Universal Health Coverage.

## Data Availability

All data generated or analyzed during this study are included in this published article.

## Abbreviations

CDoH: County Department of Health
CEC: County Executive Committee
CHMT: County Health Management Team
CPSB: County Public Services Board
HRH: Human Resources for Health
LMICs: Low and Middle-Income Countries
MCAs: Members of County Assembly
MoH: Ministry of Health
PHC: Primary Healthcare
UHC: Universal Healthcare
